# Dissimilar Changes in Serum Cortisol after Epileptic and Psychogenic Non-Epileptic Seizures: A Promising Biomarker in the Differential Diagnosis of Paroxysmal Events?

**DOI:** 10.3390/ijms25137387

**Published:** 2024-07-05

**Authors:** Flora Rider, Alexander Turchinets, Tatyana Druzhkova, Georgii Kustov, Alla Guekht, Natalia Gulyaeva

**Affiliations:** 1 Research and Clinical Center for Neuropsychiatry of Moscow Healthcare Department, Moscow 107076, Russia; 2 Department of Neurology, Neurosurgery and Medical Genetics, Pirogov Russian National Research Medical University, Moscow 117997, Russia; 3 Laboratory of Functional Biochemistry of Nervous System, Institute of Higher Nervous Activity and Neurophysiology, Russian Academy of Sciences, Moscow 117485, Russia

**Keywords:** epilepsy, epileptic seizures (ESs), psychogenic non-epileptic seizures (PNESs), hypothalamic–pituitary–adrenal (HPA) axis, cortisol, prolactin, lymphocytes

## Abstract

The hypothalamic–pituitary–adrenal axis is known to be involved in the pathogenesis of epilepsy and psychiatric disorders. Epileptic seizures (ESs) and psychogenic non-epileptic seizures (PNESs) are frequently differentially misdiagnosed. This study aimed to evaluate changes in serum cortisol and prolactin levels after ESs and PNESs as possible differential diagnostic biomarkers. Patients over 18 years with ESs (n = 29) and PNESs with motor manifestations (n = 45), captured on video-EEG monitoring, were included. Serum cortisol and prolactin levels as well as hemograms were assessed in blood samples taken at admission, during the first hour after the seizure, and after 6, 12, and 24 h. Cortisol and prolactine response were evident in the ES group (but not the PNES group) as an acute significant increase within the first hour after seizure. The occurrence of seizures in patients with ESs and PNESs demonstrated different circadian patterns. ROC analysis confirmed the accuracy of discrimination between paroxysmal events based on cortisol response: the AUC equals 0.865, with a prediction accuracy at the cutoff point of 376.5 nmol/L 0.811 (sensitivity 86.7%, specificity 72.4%). Thus, assessments of acute serum cortisol response to a paroxysmal event may be regarded as a simple, fast, and minimally invasive laboratory test contributing to differential diagnosis of ESs and PNESs.

## 1. Introduction

With the development of our knowledge about psychogenic non-epileptic seizures (PNESs), the problem of their differential diagnosis with other paroxysmal events, in particular epileptic seizures (ESs), is becoming increasingly vital. First of all, PNESs usually resemble ESs, although have no electrophysiological correlates or clinical evidence for epilepsy [1]. In other words, PNESs look like ESs clinically but lack epileptic activity during the seizure period and consist of paroxysmal events most probably facilitated by a dysfunction in emotion processing. Importantly, epileptiform EEG activity, MRI abnormalities, and neuropsychological deficits are found in several people with PNESs and especially with PNESs and epilepsy, further complicating the diagnostic process [2,3]. Thus, the difficulties of differential diagnosis of PNESs and ESs increase in cases of the coexistence of these two conditions, which is quite frequent. According to a meta-analysis [4], the mean frequency of epilepsy in patients with PNESs across all studies was 22% (95% confidence intervals (CI) 20% to 25%, range: 0% to 90%) while the mean frequency of PNESs in patients with epilepsy was 12% (95% CI 10 to 14%, range: 1% to 62%). Meanwhile, patients with a coexistence of ESs and PNESs receive more antiseizure medications and have a lower quality of life, as well as a higher risk of natural and non-natural causes of death, including a higher risk of suicide [5]. PNESs are especially common among patients with a suspected diagnosis of drug-resistant epilepsy, with the proportion of pseudo-seizures in this group reaching 20–30% [6,7]. Several patients with isolated PNESs or PNESs and ESs and a diagnosis of drug-resistant epilepsy were later found to suffer from pseudo-refractory epilepsy, even during pre-surgical evaluation.

Despite an active search for biological markers [8,9,10,11] and the development of questionnaires and interviews [12,13], the clinical experience of a physician remains important. However, in many cases, the doctor (including an emergency medical one) meets the patient immediately after or during the termination of the seizure episode and may not be able to determine the type of seizure based merely on the semiology. The “gold standard” of PNES diagnosis remains capturing a typical episode on video-EEG showing the absence of epileptiform activity immediately before, during, and immediately after the episode with a history and semiology consistent with PNESs [14]. Unfortunately, video-EEG is not available in some locations and not applicable in cases of rare events. The latency to events (ESs and PNESs) observed during prolonged EEG monitoring reaches 96 h [15]. Thus, in some patients, seizures cannot be recorded, and we still need reliable biomarkers for a quick and timely (e.g., in emergency service) diagnosis of PNESs. Discussing diagnostic techniques in the differential diagnosis of ESs and non-epileptic seizures, Cragar et al. [16] have reviewed the accuracy of possible diagnostic alternatives, including demographic and medical history variables, seizure semiology, provocative testing, prolactin levels, single photon emission computed tomography, psychological testing, and neuropsychological testing. It was concluded that any of the reviewed alternative techniques will unlikely replace video-EEG monitoring; however, the differential diagnosis of epilepsy and PNESs may greatly benefit from using combinations with complementary diagnostic tools. Serological biomarkers may support the differential diagnosis between ES and non-epileptic events [17].

An epileptic seizure is a transient occurrence of signs and/or symptoms due to abnormal excessive or synchronous neuronal activity in the brain [18] while PNESs are the result of a complex neuropsychiatric dysfunction manifested by involuntary movements and reduced self-control without any electrophysiological correlate [1,19]. Though mechanisms of PNESs are still being discussed, the “integrative cognitive model” is recognized by many researchers. According to this model, PNESs result from the activation of a learnt mental representation, like a computer program or “seizure scaffold”, often combined with concurrent physiological arousal, and contain elements of instinctive automatisms, personal illness experiences, or illness beliefs (including ESs, even witnessed) [19]. Thus, ESs may represent a type of stressful event, while PNESs signify a way to deal with stressors. In this situation stress markers might be promising candidate biomarkers for discriminations between ESs and PNESs.

Stress affects both the brain and the body. The feedback between the peripheral immune system and the brain is important for the behavioral response to stress, the cross talk between immune cells in the brain and in the periphery forming a stress susceptible individual response [20]. Lymphocytes circulating in the blood are important players supporting integrity of the immune system. Different stressors associated with changes in the functioning of immune cells affect lymphocyte count and composition [21]. The neutrophil-to-lymphocyte ratio (NLR), calculated from the complete blood count, is believed to be an indicator of systemic inflammation. Though patients with epilepsy (generalized and focal onset seizures) have a significantly higher leukocyte count in post-seizure blood, NLR could not be used to separate PNESs from ESs [22].

The stress hormone cortisol is commonly used as a universal stress biomarker [23]. However, due to the constantly high level of psychiatric comorbidities, including post-traumatic stress disorders, anxiety, and major depressive disorders in patients with epilepsy and PNESs, they are continuously exposed to high levels of cortisol [24,25,26,27]. Therefore, the hypothalamic–pituitary–adrenal (HPA) axis may become less sensitive, and stress-induced release of cortisol by the adrenals may be blunted [28].

Prolactin is a pleiotropic factor: it regulates lactation, lactotroph cell homeostasis, and maternal behavior, as well as metabolic homeostasis including body weight control, the adrenal response to stress, and many other processes in health and disease [29,30]. Serum levels of prolactin may increase as a consequence of ESs. Notably, after complex partial seizures or generalized tonic–clonic seizures, serum prolactin transiently augments in approximately 60% of cases, while interictal epileptic discharges from the temporal lobe may induce long-term modifications of the hormone release [31]. The release of prolactin is induced by the spreading of epileptic activity from the temporal lobe to the HPA axis. Prolactin usually fails to rise after psychogenic seizures; therefore, postictal prolactin levels can be potentially used to differentiate between epileptic and psychogenic seizures. However, in repetitive seizures, postictal release of prolactin may decrease, while effects of chronic epileptic discharges and anticonvulsants on prolactin release are moderate [32]. In male and female patients, elevations of prolactin in blood were seen immediately and at 20 min after generalized tonic–clonic seizures; as a rule, prolactin levels return to normal values within 1 h [33]. The authors regard serum prolactin as a reliable confirmatory test in the presence of a seizure, but not that effective as a screening test for suspected seizures.

Repeated efforts to employ serological stress-responsive biomarkers to distinguish between ESs and PNESs have been made during the last almost four decades. Several attempts to use prolactin and cortisol markers for differential diagnosis of ESs and PNESs were performed in the late 1980s; however, only very small groups of patients were studied [34,35]. To our knowledge, no studies have been conducted on larger groups of patients, including patients with a combination of ESs and PNESs. As well, the circadian pattern of ES and PNES occurrence has not been considered in most studies. In this study, we focused on the biomarkers available in most health facilities and routinely used in laboratory *practices*. We aimed to study the response of cortisol and prolactin in the blood of patients with PNESs and ESs during 24 h after the paroxysmal event and substantiate an additional stress-responsive biomarker for differential diagnostics of PNESs and ESs even in cases of coexistence of these types of events in the same patient.

## 2. Results

### 2.1. Characteristics of Patients at Admission

The patients in the groups studied did not differ in age or main socio-demographic characteristics (Table 1).

Regarding clinical data (Table 1), among the patients of the PNES group, significantly more patients with anxiety disorders (92% versus 48% in the ES group; χ^2^ test, *p* < 0.001) were identified. However, no significant difference in the rate of patients with current depressive episodes (cDEs) could be revealed between the ES and PNES groups. Importantly, there were patients without psychiatric conditions (38%) in the ES group but no such patients in the PNES group (χ^2^ test, *p* < 0.001). Significantly fewer patients had two or more mental disorders in the ES group (38%) as compared with the PNES group (62%; χ^2^ test, *p* = 0.042).

Interestingly, all the patients in the ES group had a long history of seizures (median 16 years, IQR 10–28 years) compared to those in the PNES group (median 3 years, IQR 1.8–6 years, Mann–Whitney U-test, *p* < 0.001). In the PNES group, there was also a trend towards an older age at paroxysmal events onset (Mann–Whitney U-test, *p* < 0.001). Among patients with temporal lobe epilepsy (thirteen patients, 45%) only three did not have any comorbid mental disorders, while eight had two or more conditions.

**Figure 1:**
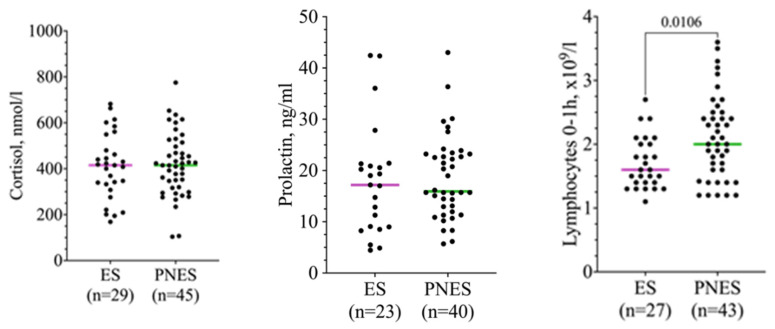
Scatter plots showing the distribution of basic cortisol, prolactin, and lymphocyte levels in patients with epileptic seizures (ES, n = 29) and psychogenic non-epileptic seizures (PNES, n = 45). The colored line represents the mean. The difference between the baseline levels of lymphocytes in patients with ESs and PNESs is statistically significant (*p* = 0.01, Mann–Whitney U-test).

Serum cortisol and prolactin levels the next morning after admission (base) did not differ between the two groups (cortisol: ES, 410 ± 143.2; PNES, 417.2 ± 137.1 nmol/L, Student’s unpaired t-test, *p* = 0.8; prolactin: ES, 17.8 (9–21.3); PNES = 15.9 (12.2–23.2) ng/mL, Mann–Whitney U-test, *p* = 0.4) (Table 1). The distribution of individual values is shown in Figure 1. Noteworthy is that the level of lymphocytes at admission was higher in patients with PNESs (ES, 1.6 (1.4–2.0); PNES, 2.0 (1.6–2.5) × 109/L, Mann–Whitney U-test, *p* = 0.01).

### 2.2. Circadian Differences in ES and PNES Occurrence and Response of Serum Cortisol, Prolactin, and Lymphocytes to Different Paroxysmal Events

**Figure 2:**
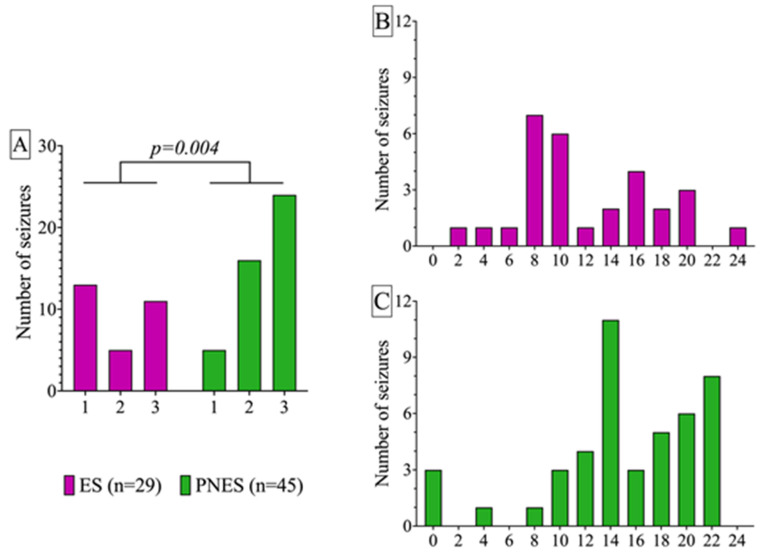
Frequency distribution of seizure occurrence time in groups with epileptic seizures (ESs) and psychogenic non-epileptic seizures (PNESs). (**A**) Distribution of the episodes by periods of the day: 1—morning, 2—day, 3—evening–night. PNESs were significantly skewed towards the evening–night time group (*p* = 0.004, 3 × 2 χ^2^ test). (**B**,**C**) Distribution by hours during the day in the epileptic seizures ES (**B**) and PNES (**C**) groups. The occurrence rate of seizures at different periods of the day for ESs and PNESs, respectively, were as follows: morning, 13 and 5; day, 5 and 16; evening–night, 11 and 24.

The circadian distribution of seizure occurrence is shown in Figure 2. In the PNES group, seizures occurred significantly more often in the evening, while in the ES group they tended to occur at night and in the morning (χ^2^-test, *p* = 0.004).

**Figure 3:**
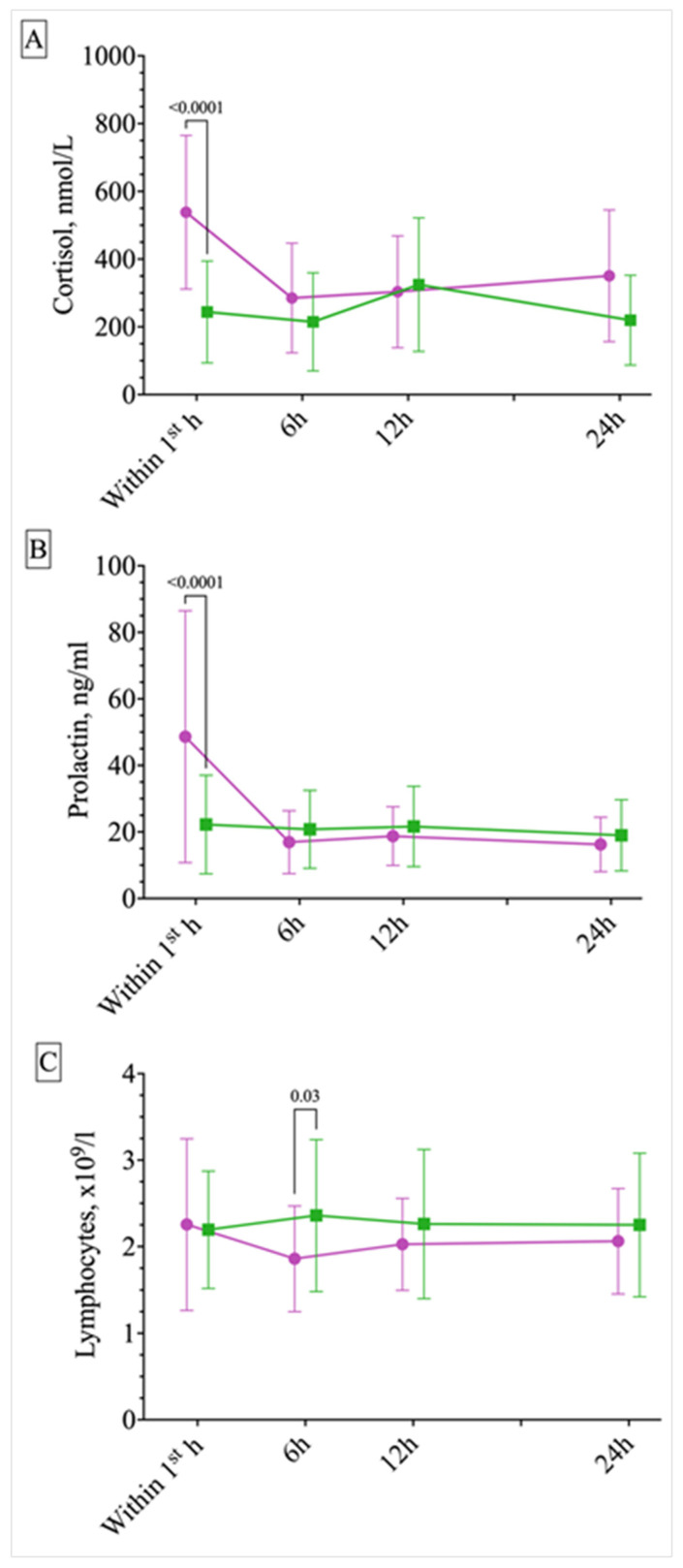
Changes in serum cortisol (**A**), prolactin (**B**), and lymphocyte (**C**) levels after a paroxysmal episode. Green symbols represent psychogenic non-epileptic seizures (PNESs), purple symbols represent epileptic seizures (ESs). M ± SD are presented. There are statistically significant differences in cortisol (**A**) and prolactin (**B**) levels between the two groups within the first hour after the seizure (*p* < 0.001, Tukey’s test). Within one hour following an ES, a transient increase in the number of lymphocytes occurs, as compared to baseline levels (**C**). The lymphocyte count after ES returns to baseline by the 6 h time point where it is lower than the lymphocyte count in the PNES group (*p* = 0.03, Tukey’s test).

**Figure 4:**
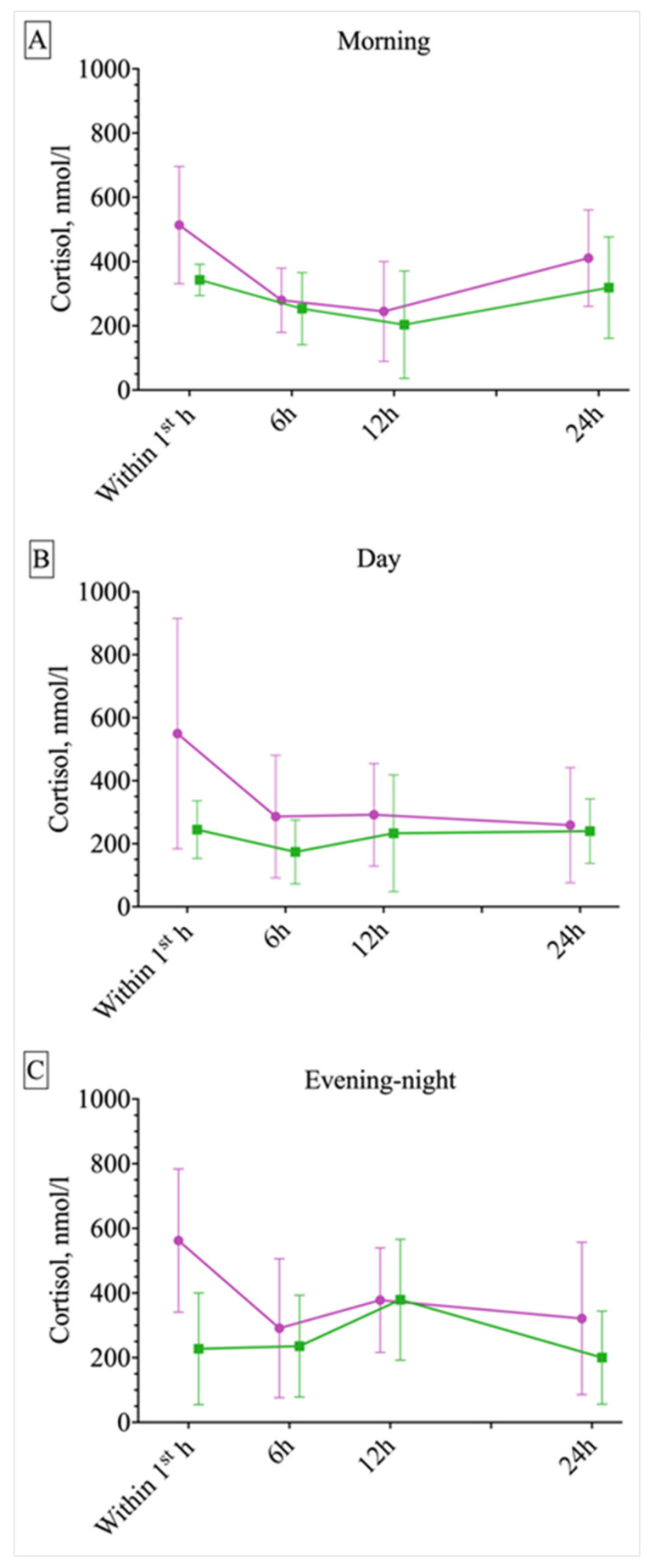
The response of serum cortisol to epileptic seizures (ESs) or psychogenic non-epileptic seizures (PNESs) depending on the time of paroxysmal event onset: morning (**A**), day (**B**), evening–night (**C**). Green symbols represent psychogenic non-epileptic seizures (PNESs), purple symbols represent epileptic seizures (ESs). Points represent the mean values, and vertical bars represent standard deviations. The circadian fluctuations in cortisol levels were attenuated in both groups; however, an acute increase in cortisol levels during the first hour following the seizure was revealed in the ES group.

The data on the time course of blood indices after a paroxysmal event are presented in Figure 3 (summary data) and Figure 4 (separately, depending on the time of seizure occurrence). Cortisol levels within the first hour after the event significantly differed between the ES and PNES groups, with the acute cortisol level being more than twice as high in the ES patients (*p* < 0.001, Mann–Whitney U-test); this difference leveled out at subsequent time points (Figure 3A).

Taking into account that seizures in patients with PNESs occurred more often in the evening, which potentially could influence the overall trend for cortisol, an assessment of the time course of serum cortisol levels in each group was additionally calculated for morning, day and evening–night time periods separately (Figure 4). It can be seen that circadian fluctuations in cortisol levels are smoothed out in both groups; however, in the ES group an acute increase in cortisol levels was revealed (more obvious in the evening–night subgroup, where the 12 h point corresponds to the morning peak of serum cortisol, RM-ANOVA, Tukey post hoc test, *p* = 0.02). In the PNES group, the cortisol level does not change significantly (though circadian difference may be observed in the evening–night subgroup, RM-ANOVA, Tukey post hoc test, *p* = 0.007). Despite the obvious variations in cortisol levels depending on cortisol circadian rhythm, the acute response induced by the seizure is divergent in ESs and PNESs, indicating the importance and potential usefulness of this phenomenon (Figure 4).

We also assessed the changes in prolactin levels in the blood serum of patients with ESs and PNESs. Outlier detection was performed using GraphPad Prism built-in algorithm ROUT (Q = 5%). After excluding outliers, the studied cohorts included 23 patients (11 males) in the ES group and 40 patients (12 males) in the PNES one. No statistically significant differences in gender or age between the newly formed groups (χ^2^-test, *p* = 0.1) could be detected. A significant acute increase (*p* < 0.001) in prolactin levels within an hour after the seizure followed by a decrease (*p* < 0.001) to baseline levels was revealed in the ES group, while no significant change in prolactin levels was evident in the PNES group. Prolactin levels were significantly different in the two groups within an hour after the attack (*p* < 0.001, two-way ANOVA, post hoc Tukey test).

The changes in the level of lymphocytes were assessed after a paroxysmal event in both groups, excluding four identified outliers (Figure 3C). As noted above, at admission, lymphocyte levels were significantly higher in patients with PNESs (Table 1). During the acute post-seizure period the number of lymphocytes in patients with ESs was higher than 6 hours after the seizure (acute post-seizure period as compared to the 6 h time point, *p* = 0.0187, one-way RM-ANOVA, post hoc Tukey test). There was no difference between the ES and PNES groups at the first (acute) time point, but at the 6 h time point the lymphocyte count was higher in patients with PNESs (*p* = 0.03, two-way ANOVA, post hoc Tukey test).

Calculations using a two-way ANOVA showed that in the cohort studied gender was not a significant factor influencing either the basal levels or seizure-induced changes in the biomarkers studied. No interaction between time and gender could be revealed.

### 2.3. Correlations

**Figure 5:**
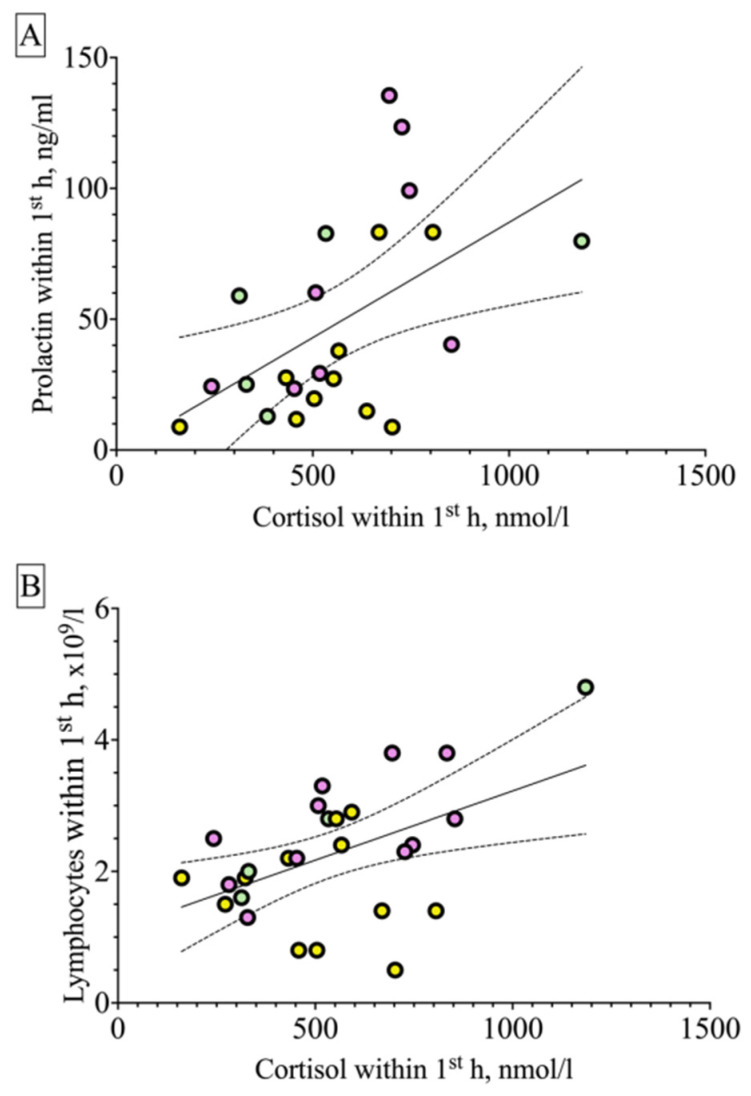
Correlations of serum cortisol level and prolactin level (**A**), and cortisol level and lymphocyte count (**B**) during the acute period after seizures in patients with epileptic seizures (ESs), assessed by the Spearman method. The solid line represents the best approximation, the dashed lines represent the 95% confidence interval. Yellow symbols, morning seizures; green symbols, daytime seizures; purple symbols, evening–night seizures.

Taking into account similar responses of blood serum cortisol and prolactin in the patients of the ES group, a correlation analysis of these two indices was conducted, considering excluded outliers (n = 23). A highly significant correlation, r = 0.55 (Spearman’s correlation, 95% confidence interval is 0.17–0.79, *p* = 0.006), was found, most probably indicative of a common pathogenetic HPA axis-related mechanism responsible for increasing the level of both hormones during ESs (Figure 5A). A moderate correlation, r = 0.4 (Spearman’s correlation, 95% confidence interval is 0.007–0.68, *p* = 0.04), was found between the level of lymphocytes in the blood and the level of cortisol in the serum in patients with ESs (Figure 5B). No correlations were observed in the PNES group.

### 2.4. ROC Analysis

To create a prognostic model, ROC analysis was performed for the cortisol and prolactin within an hour after the event. Based on the values of sensitivity, specificity, and their sum, a graph was built to determine the cutoff points. In terms of specificity, the cutoff points were identified for cortisol: >300 nmol/L (specificity 70%), >400 nmol/L (specificity 80%), >500 nmol/L (specificity 90%), and >600 nmol/L (specificity 100%). The odds ratio for 1 nmol/L of serum cortisol was 1.008 (95% CI 1.004–1.013), i.e., with an increase in cortisol levels of 100 nmol/L, the probability of an epileptic etiology of a paroxysmal event increases 2.2 times.

**Figure 6:**
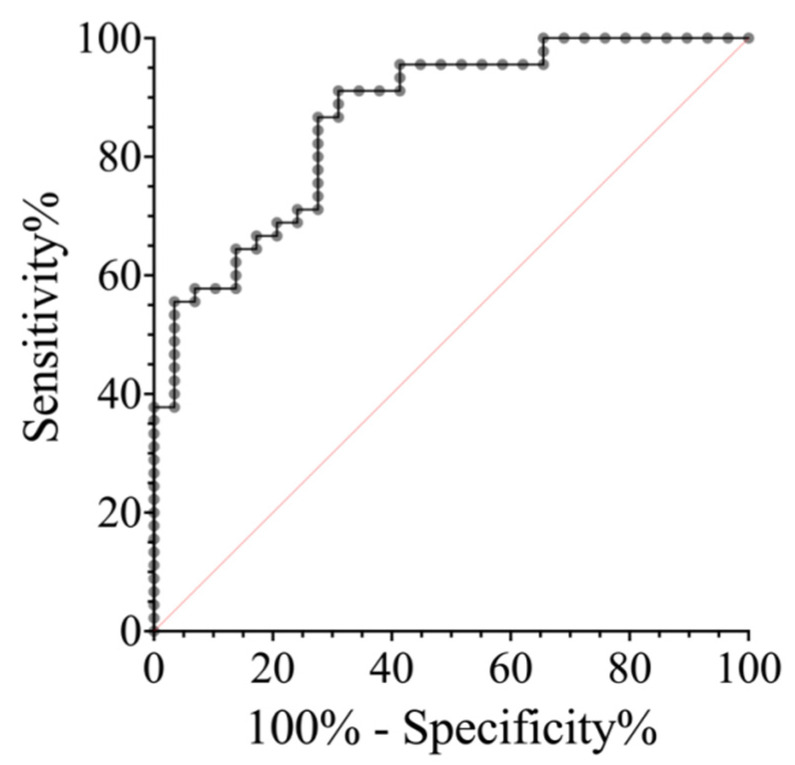
ROC analysis of the serum cortisol levels during the first hour after the seizure.

The representation of the accuracy of paroxysmal event type discrimination based on cortisol levels assessed using ROC analysis is presented in Figure 6. The area under the curve (AUC) was 0.865 and the prediction accuracy at the cutoff point of 376.5 nmol/L was 0.811 (sensitivity 86.7%, specificity 72.4%).

## 3. Discussion

PNESs are attacks that resemble ESs but lack epileptiform brain activity, and that is why they are frequently misdiagnosed. In patients referred to epilepsy centers, PNESs are the most widespread non-epileptic disorder. Though it is widely believed that PNES events are harmless, the death rate of PNES patients is reported to be comparable with drug-resistant epilepsy [36].

Though, externally, PNESs look a lot like epileptic seizures, the cause of PNESs is assumed to be psychogenic. PNESs are closely linked with psychological distress. An abnormal coping strategy demonstrated by PNES patients manifests in their increased tendency to dissociate [37]. The anomalously strong functional connectivity in PNES patients is regarded as a neurophysiological correlate for the underlying psychoform and somatoform dissociation mechanism where emotions affect executive control and induce altered motor function resulting in seizure-like episodes. Psychiatric disorders are more common in patients experiencing PNESs as compared with ESs [38]. Though psychopathology levels are generally elevated in patients with both PNESs and ESs, patients with PNESs report higher rates of trauma and neglect, as well as an increased prevalence of insecure attachment [39]. Emotional responses of PNES patients to affective pictures suggest intense emotional experience and diminished positive emotional behavior [40]. Levels of anxiety and depression are usually higher in patients with PNESs as compared with ESs, and interpersonal problems are much more closely associated with anxiety and depression. Systematic reviews and meta-analyses not only demonstrate a higher prevalence of comorbid depression in patients with PNESs as compared to patients with epilepsy, but also suggest differences in the expression and possible causes of depression between these groups [41]. A meta-analysis supports an important, although not essential, role for panic and hyperventilation in the pathogenesis of PNES events [42]. One of the strengths of our study is the fact that all patients were examined by qualified psychiatrists, who revealed a higher prevalence of anxiety disorders in the group of patients with PNESs compared with patients with epilepsy (Table 1). Though the PNES group examined in the present study, indeed, demonstrated a higher rate of anxiety, we did not reveal a higher rate of depression (Table 1). Unlike the ES group containing more than one third of patients without psychiatric conditions, all patients in the PNES group demonstrated psychiatric disturbances, and the occurrence of patients with two or more mental disorders in the PNES patients was dramatically higher than compared with the ES group. From our perspective, this makes the revealed absence of cortisol increase after PNESs even more important. Due to the relatively large cohort studied, we could demonstrate that, despite a significantly higher frequency of PNESs as compared to ESs in the evening–night time groups (*p* = 0.004; it can be explained by the absence of familiar medical staff at this time and a subsequent increase in the anxiety of patients), the general trend in the pattern of cortisol response remains the same irrespective of the time of the day.

Patients with PNESs demonstrate a higher-than-average prevalence of anatomical brain anomalies detected by structural changes in brain MRI. The growing body of data suggests that PNESs are not a “medical mystery” but rather a disorder with physical defects in the brain [43]. An anomalous connectivity between brain areas involved in emotional processing and cognitive integration systems, and motor and premotor regions may explain the paroxysmal events in patients with PNESs. fMRI hyporeactivity to psychological stress in PNES patients, along with greater emotion–motor–executive control network resting state functional connectivity, when compared to healthy controls, suggest a dysregulation in stress response circuitry in PNESs [44]. However, so far neuroimaging investigations of PNESs have not provided a simple and reliable approach to discriminate between them and ESs.

PNESs are predominantly seen in women (which corresponds to the gender ratio in patients enrolled in the present study). This may be associated with existing sex-dependent intrinsic functional connectivity differences in brain areas responsible for emotional and cognitive processing and differences in the vulnerability of men and women to physical or emotional trauma [45]. Gender-dependent intrinsic brain connectivity differences may be a reason for the prevalence of females among patients with PNESs. However, in our study, none of the parameters studied were gender-dependent.

There have been numerous attempts to elaborate realistic approaches to differential diagnostics of ESs and PNESs using diverse techniques. However, most of the studies have reported important phenomena uncovering specific features of physiological and molecular mechanisms of these types of paroxysmal events, yet they could not be converted into useful, simple, and feasible methods to discriminate between ESs and PNESs. Video-EEG monitoring is the “gold standard” for ES and PNES diagnosis, yet not all patients experience convulsive episodes during video-EEG sessions. An effective tool in supporting PNES diagnosis without a video-EEG recording may be EEG connectivity and machine learning methods using interictal recordings [46]; however, it is hardly affordable for emergency service physicians. Multidimensional psychopathological profile differences have been revealed between patients with PNESs and ESs [47]. Patients with PNESs report more psychopathological features in general, and demonstrate particularly high occurrences of childhood trauma, dissociation, and depression. Indeed, PNESs usually reflect poor motor and sensory function caused by psychological responses to stressful experiences. According to the integrative cognitive model, PNESs represent an automatic reaction produced by vague memory and are perceived as unmanageable and superfluous [19]. Importantly, this model suggests that a PNES event is induced by an external or internal cue. Schneider et al. [48] have demonstrated a unique preictal behavior before PNES events with motor manifestations. Since the specific PNES preictal behavior consists mainly of inactivity, the authors believe that PNESs represents a freeze reaction or a reconstruction of a freeze reaction. Notably, freezing in experimental animals is regarded and used in many routine behavioral tests as an undeniable sign of fear.

The research on the molecular mechanisms of PNESs remains limited. A number of genes and hormones have been found to be associated with PNESs and potentially involved in the disease pathogenesis network, including proopiomelanocortin, neuropeptide Y, cortisol, norepinephrine, and brain-derived neurotrophic factor (BDNF). Signal transduction cascades and molecules, in particular, Janus kinase/signaling transducer and activator of transcription (JAK-STAT), JAK, signaling through growth hormone receptor, phosphatidylinositol 3-kinase/protein kinase B (PI3K/AKT), and neurotrophins are associated with PNESs [36]. Notably, sharing mechanisms of signal transduction may explain the association of depression, anxiety, schizophrenia, and alcohol-related disorders with PNESs.

A number of blood markers potentially associated with ESs and PNESs have been studied. Postictal blood tests are important for discovering the causes of symptomatic seizures due to endocrine, metabolic, toxic, or infectious factors [49]. Metabolic markers such as ammonia and lactate may have diagnostic potential for postictal blood tests. Serum lactate can be a valuable tool to differentiate generalized tonic–clonic seizures from other forms of transient loss of consciousness, but it has not proven valuable in distinguishing ESs from PNESs, should not be used as an absolute diagnostic tool, and should be interpreted along with proper clinical context [50]. According to the data of a retrospective study and meta-analysis of 1300 patients, elevated serum levels of creatine kinase have been associated mainly with ESs with regard to non-epileptic events [51]. Elevations in the creatine kinase levels are frequent after generalized tonic–clonic seizures and display high specificity but moderate sensitivity.

The postictal serum levels of ubiquitin C-terminal hydrolase (UCHL-1), a neuronal biomarker, and S100-B, a glial biomarker, were assessed in an attempt to differentiate ESs from PNESs [11]. The serum UCHL-1 level could be potentially used for this purpose (sensitivity 72%, specificity 59%), while the serum S100-B level had a lower value compared to UCHL-1 (AUC 0.68 for UCHL-1 vs. 0.59 for S100B). These indices could be used in future studies to understand the mechanisms underlying seizures and form the basis for an additional diagnostic tool to differentiate ESs from PNESs. Since the serum neurogranin level is high in patients who have experienced an ES, but not PNESs, this marker can be also considered for potential use in the differential diagnosis of ESs and PNESs [52].

Attempts have been made to discriminate immune responses in blood before and after ESs and PNESs [53]. Interictal levels of interleukin-6 (IL-6) were increased in serum samples from patients with video-EEG-verified temporal or frontal lobe epilepsy (TLE or FLE, respectively) or TLE + PNESs as compared to healthy controls, while patients with PNESs did not demonstrate an increase in IL-6. Postictal IL-6 levels increased transiently within hours in TLE but not in FLE patients. The postictal to interictal ratio of five additional immune factors were also increased in TLE patients only. Thus, immune factors have the potential to be future biomarkers for ESs and the heterogeneity among different ESs and PNESs may be revealed in peripheral blood samples independently of comorbidities [53]. However, though monitoring the systemic immune reaction is an attractive diagnostic approach to differentiate between ESs and PNESs, it is not in practical use so far. Tan et al. [54] validated peripheral cell ratio and lactate score for differentiating status epilepticus from prolonged PNESs and showed the differing post-event time profiles of lactate levels versus neutrophil count, the systemic immune-inflammation index (SII, neutrophils × platelets/lymphocytes), and the systemic inflammation response index (SIRI, neutrophil count × monocyte count/lymphocyte count). In our study, blood lymphocyte count did not appear to be a suitable index to differentiate ESs from PNESs.

Neuropeptide Y has been associated with resilience to stress. Compared with female controls, PNES patients had lower neuropeptide Y levels; this may confer higher vulnerability to exhibit seizure-like symptoms [55]. Miani et al. tried to construct a predictive model in order to detect PNESs from serum hormone levels, detached from an evaluation of patients’ convulsive episodes [56]. They found that levels of neuropeptide Y and adrenocorticotropic hormone (ACTH) were the optimal combination of predictors, with over 90% accuracy (AUC = 0.980).

Recent studies indicate hyperactivity of the HPA axis and elevated levels of glucocorticoids in TLE patients [57]. In these patients, stress is a usually reported trigger of seizures, and stress-related psychopathologies (depression and anxiety) are highly prevalent. Comorbid TLE and depression associated with dysfunction of the HPA axis are based on common molecular and cellular mechanisms, including the dysfunction of glucocorticoid receptors, neurotransmitters, and neurotrophic factors, and the development of neuroinflammation, leading to neurodegeneration and loss of hippocampal neurons, as well as disturbances in neurogenesis in the subgranular neurogenic niche and formation of aberrant neural networks [58]. Important clinical implications of the HPA axis for seizure control are well established [59]. Women with more frequent seizures have demonstrated an increase in cortisol and a decrease in dehydroepiandrosterone sulfate levels, hormonal changes obviously relevant in seizure control [60]. Cortisol fluctuations relate to interictal epileptiform discharges in stress-sensitive epilepsy, at least partially, due to the effects of cortisol on neuronal excitability [61,62]. People with stress-sensitive epilepsy demonstrate a whole-brain neuronal response to cortisol dissimilar to that of patients with non-stress-sensitive epilepsy.

Regardless of stress, cortisol is released in hourly pulses, and circadian cortisol is positively related to the occurrence of epileptiform discharges in people with stress-sensitive seizures (not those with stress-insensitive seizures). The association between cortisol levels and frequency of interictal epileptiform discharges in people with stress-sensitive seizures confirms that cortisol influences disease activity in epilepsy, under basal conditions as well [63]. In epilepsy patients, the serum concentrations of cortisol and ACTH during sleep seizures are associated with preictal and ictal EEG alterations [64]. Most of the observed circadian distributions of epileptiform discharges could be explained by cortisol time course or sleep stage transition, or a combination of both [65].

Many patients with epilepsy or PNESs experience high levels of stress [66]; however, coping with stress may be different in these groups. The association of epilepsy with stress hormone levels and the involvement of stress in epileptogenesis and seizure precipitation is a basis to consider epilepsy a chronic stress model [58,67]. PNESs are events that appear epileptic but are believed to have a psychological origin. Several studies have pointed to associations of PNESs with psychological trauma; however, not many studies have examined the connections with neurobiologic stress systems, such as the HPA axis and cortisol. Bakvis et al. [68] assessed some important HPA axis functions in patients with PNESs and correlated them to trauma history. This group reported that patients with PNESs showed significantly increased basal diurnal cortisol levels as compared to healthy controls. Patients reporting sexual trauma showed a trend toward higher cortisol levels as compared to patients without a reported sexual trauma. PNES patients demonstrated basal hypercortisolism suggesting that HPA axis activity is an important neurobiologic marker for PNESs [68]. In our study basal (at admission) levels of blood cortisol were similar in ES and PNES groups (Table 1). Since mild hypercortisolism was shown in patients with epilepsy and/or depression as well [27], this result not only confirms the previous data, but also supports the common high stress load in patients with PNES and ESs.

Cortisol, the main glucocorticoid hormone in humans and an important component of adaptive responses to stressogenic stimuli, shows a typical stress response as secretion from adrenals and a transient elevation in blood. Anti-inflammatory effects of glucocorticoids are widely used in clinical practice, while their pro-inflammatory effects are believed to underlie neurodegeneration. This is particularly critical for the lymbic system [69]. Glucocorticoid hormones ensure the coordinated functioning of key components and mechanisms of hippocampal plasticity, inducing diverse regulatory mechanisms mediated by their receptors [70]. In a case-control study, the association between the glucocorticoid receptor gene (NR3C1) rs41423247 polymorphism and PNESs was investigated [71]. Patients with PNESs and those with major depression disease were significantly more often G allele carriers in rs41423247 as compared with healthy controls. A significant association between CG genotype and PNESs was demonstrated, however the possibility of confounding effects of depression could not be excluded.

In a small-scale study (six patients with ESs and five patients with PNESs) in the late 1980s, Rao et al. [35] showed that ESs but not PNESs are accompanied by the simultaneous elevation of serum pituitary hormones and cortisol levels. The simultaneous elevation of serum prolactin, thyrotropin, growth hormone, and cortisol pointed to a central stimulation of the HPA axis during ESs, but not during seizures of psychogenic origin. In the present study, much larger groups (ES 27, PNES 45) were studied. Importantly, in the PNES group, seizures occurred predominantly in the evening, while in the ES group mostly at night and in the morning. Cortisol levels within the first hour after seizure demonstrated a significant acute elevation in the ES group and an opposite trend in the PNES group. Additional calculation of cortisol levels separately for morning, day, and evening–night time periods (to exclude potential circadian effects) showed that circadian cortisol fluctuations in levels were flattened in both groups, but both the acute increase in cortisol in the ES group and the lack of cortisol response in patients with PNESs were evident during all day periods studied. In other words, the differential effect of ESs and PNESs on cortisol levels did not depend on cortisol circadian rhythm. A strong correlation between acute cortisol and prolactin levels was revealed in the ES group specifically. Our data confirm the results reported by Molaie et al. [72] that postictal elevation of plasma prolactine is a specific phenomenon related to seizure discharges; however, failure of such a rise does not exclude partial seizures. For the differentiation of ESs from PNESs, several studies have shown that elevated serum prolactin may be highly predictive of either generalized tonic–clonic or complex partial seizures [73]. Prolactin testing may help to differentiate ESs from PNESs, and is associated with rather high specificity but only moderate sensitivity [49]. In this present study, the patterns of acute response of prolactin to ESs and PNESs were in general similar to the response of cortisol. However, heterogeneous distribution of acute prolactin levels (the range of the acute prolactin levels was extremely high) discourages the use of this biomarker in differentiating ESs and PNESs. Notably, significant correlations exist between cortisol and prolactin levels as well as cortisol and lymphocyte count in the ES group confirming the involvement of an intricate stress response mechanism in epileptic ictogenesis.

In this present study we have shown that acute postictal cortisol levels can be potentially used in the differential diagnosis of ESs and PNESs. Cutoff points were determined for the specificity from 70 to 100%, and ROC analysis showed an AUC of 0.865 and a prediction accuracy at the cutoff point of 376.5 nmol/L 0.811 (sensitivity 86.7%, specificity 72.4%). Thus, acute cortisol levels after a paroxysmal event are a reliable laboratory marker for differentiation between ESs and PNESs.

## 4. Materials and Methods

### 4.1. Subjects, Procedure, and Instruments

This study was conducted between February 2021 and February 2023 in the Moscow Research and Clinical Center for Neuropsychiatry. All procedures in studies involving human subjects have been conducted in accordance with a set of ethical principles presented in the Seventh Revision of the Declaration of the World Medical Association [74]. The studies were conducted in accordance with local legislation and institutional requirements; the protocol of the study was approved by the Research Ethics Committee of the Moscow Research and Clinical Center for Neuropsychiatry (approval #42, 19 August 2019) with informed consent obtained from all subjects.

Adult patients with epilepsy and/or suspected PNESs were evaluated on the day of admission by psychiatrists experienced in both mental disorders and epilepsy. Diagnosis of mental disorder was based on ICD-10 criteria, while cognitive function was assessed using the Mini-Mental Scale Examination (MMSE) [75]. Patients with schizophrenia spectrum disorders, organic psychotic disorders, severe comorbid somatic or neurological disorders, and cognitive impairment (MMSE score below 24) were excluded.

Epilepsy was diagnosed according to ILAE criteria [18] by two experienced epileptologists on the basis of medical history, neurological examination, instrumental diagnostic methods, and analysis of seizure video recordings. All patients with ESs and/or suspected PNESs, both with motor manifestations, underwent video-EEG monitoring (including ictal recording) to confirm them, and only those with ESs or PNESs, captured on video-EEG recording (documented PNESs, [76]), who signed informed consent for repeated blood tests, were included.

According to the criteria described above, 74 patients over 18 years with ESs (n = 29) and PNESs, both with motor manifestations (n = 45) and captured on video-EEG monitoring, were included. ESs included 15 focal to bilateral tonic–clonic seizures (8 in females, 7 in males) and 14 focal motor and non-motor onset seizures with impaired awareness (11 in females, 3 in males). The patients were not treatment naïve and received appropriate medications (treatment as usual) prescribed by an experienced epileptologist and/or psychiatrist. Among the patients with non-epileptic seizures, 19 also had ESs and took antiseizure medications. All patients with epileptic seizures were diagnosed with focal epilepsy, while 13 patients (45%) had temporal lobe epilepsy.

The patients of both groups were further divided into subgroups depending on the time of the seizure onset, considering that the level of cortisol in the serum has a diurnal pattern of fluctuation [77]. Accordingly, three subgroups were formed: morning (6:00 a.m.–10:59 a.m.), day (11:01 a.m.–3:59 p.m.), and evening–night (4:00 p.m.–5:59 a.m.).

### 4.2. Blood Sampling, Assessment of Hormones, and Hemogram

Biochemical and hormonal indices were measured in blood serum obtained from fasting morning venous blood. Samples were collected in gel/clotting activator S-Monovette tubes and centrifuged at 2000× *g* for 10 min at 8 °C in an Allegra X-30R Centrifuge (Beckman Coulter, Brea, CA, USA). Cortisol and prolactin levels were measured in blood serum by competitive enzyme immunoassay using appropriate kits (Beckman Coulter, Brea, CA, USA) and an ACCESS^®^ 2 immunoassay automated analyzer (Beckman Coulter, Brea, CA, USA). Complete blood count with differential white blood cell count (CBC with diff) and hemogram were performed on an automated analyzer LH-500 (Beckman Coulter, Brea, CA, USA).

Blood collection was performed the next morning (6:00 a.m.–10:59 a.m., fasting) after the admission (base) and repeated four times after the paroxysmal event: during the first hour (within 1 h), and 6, 12, and 24 h after it.

### 4.3. Statistical Analysis

Statistical analysis was performed in STATISTICA for Windows ver. 12 (StatSoft Inc., Tulsa, OK, USA) and GraphPad Prism ver. 9.4.1 (GraphPad Software, USA). For the assessment of normality, the Shapiro–Wilk test was used. Results are presented as mean ± standard deviation (M ± SD) or as median and interquartile range [Me(IQR)] for non-normally distributed data. Unpaired Student’s t-test and Mann–Whitney U-test were used to compare two groups. Within the same group, the difference between different time points was evaluated with repeated measures one-way ANOVA. Multiple comparisons were made using a post hoc Tukey test. To reveal the relationship between variables, correlation analysis was performed with the calculation of the Spearman rank correlation coefficient (r). ROC analysis was carried out using the Wilson–Brown method, and a 95% confidence interval was set. Outliers were determined in GraphPad Prism using the built-in ROUT algorithm (Q = 5%). Differences were considered significant at *p* < 0.05.

## 5. Conclusions

Differentiating PNESs and ESs can be difficult clinically, even when expert clinicians have video recordings of seizures and it is much trickier in situations without video-EEG monitoring. The differential diagnosis of ESs from PNESs remains a challenging situation, first of all for emergency service. As such, reliable quantitative tools are needed to help differentiate ESs from PNESs and make an early, accurate diagnosis. A biochemical analysis may be a promising source of potential biomarkers for differentiating ESs and PNESs. Sundararajan et al. [78], in a systematic review, state that prolactin is elevated in ESs but not PNESs, although it shows low diagnostic sensitivity; postictal cortisol and creatine kinase are nonspecific, while other miscellaneous biomarkers show no conclusive evidence of utility. The authors conclude that no single biomarker successfully differentiates PNESs from ESs; in fact, PNESs are only diagnosed via the negation of ESs. Our data clearly show that acute postictal cortisol level in blood serum is unambiguous with respect to discrimination between ESs and PNESs and is an appropriate laboratory marker that can help clinicians determine whether an unwitnessed event is more likely to be epileptic or non-epileptic. This approach is simple and reliable, though additional studies should be performed to implement the method in clinical practice.

Though attempts to use cortisol response to a seizure to distinguish between ESs and PNESs is not novel (an analysis of previous studies was performed in this paper), we were the first to perform a much larger scale study than has been performed before and to confirm that changes in cortisol levels induced by ESs and PNESs are reliably different and suitable as an additional approach to differential diagnosis. No doubt that video-EEG and clinical manifestations remain the main method and gold standard, but cortisol changes may be a useful supplementary approach in urgent situations when video-EEG is not available. In addition, cortisol response to a seizure episode is important for understanding differential pathophysiological mechanisms of ESs and PNESs and forms a good platform for further studying these mechanisms. The patterns of cortisol response to ESs or PNESs are opposite because of the dramatically different natures of these seizures. The response of cortisol, a non-specific marker of stress response, becomes specific when a differentiation between two types of seizures should be made. While an ES induces a routine stress response, a PNES shows a trend of decrease in cortisol level, suggesting an instant temporary blockage of cortisol release from the adrenals. Revealing the physiological mechanism of this phenomenon will expand our understanding of the cause and neuroendocrine basis of PNESs.

## Figures and Tables

**Table 1:** Characteristics of patients at admission.

Parameter/Group	ES (n = 29)	PNES (n = 45)	*p*-Value
*Socio-demographic data*			
Age, years, M ± SD	41.6 ± 13.8	36 ± 13.8	0.08 ^2^
Females, n (% of patients in the group)	18 (62%)	33 (73%)	0.3 ^3^
Education, n (%) Secondary Higher	21 (72%) 8 (28%)	34 (76%) 11 (24%)	0.76 ^3^
Employment, n (%) Student/employed Unemployed	9 (31%) 20 (69%)	8 (18%) 37 (82%)	0.19 ^3^
Marital status, n (%) Single/widowed/divorced Married/in a relationship	19 (66%) 10 (34%)	35 (78%) 10 (22%)	0.25 ^3^
*Clinical data*			
Current depressive episode, n (%)	15 (52%)	24 (39%)	0.89 ^3^
Anxiety disorders, n (%)	14 (48%)	42 (92%)	***<0.001*** ^3^
MMSE, M ± SD	28 ± 2.9	28 ± 1.9	1.0 ^1^
Period of paroxysmal events registration, years, Me (IQR)	16 (10–28)	3 (1.8–6)	***<0.001*** ^2^
Age of paroxysmal events onset, years, Me (IQR)	18 (11–37)	27 (18–40)	***<0.001*** ^2^
Frequency of paroxysmal events, n (%) 1–2/year 3–11/year More than 12/year	3 (10%) 8 (28%) 18 (62%)	0 8 (18%) 37 (82%)	***0.041*** ^3^ (*3 × 2* χ^2^ test)
*Hemogram*			
While blood cells, WBCs, 10^3^ µL	5.46 ± 1.5	6.18 ± 1.41	***0.04*** ^1^
Lymphocytes, %, Me (IQR) 10^3^ µL, Me (IQR)	34.8 (28.7–41) 1.6 (1.4–2.0)	38.2 (29.5–44)/ 2.0 (1.6–2.5)	***0.01*** ^2^
Neutrophils, NEI, %, M ± SD 10^3^ µL, M ± SD	53.43 ± 9.06 2.93 ± 0.93	53.02 ± 10.43 3.47 ± 1.43	0.08 ^1^
Monocytes, MO, %, M ± SD 10^3^ µL, M ± SD	8.86 ± 2.74/ 0.47 ± 0.14	8.31 ± 2/ 0.49 ± 0.13	0.32 ^1^
Hemoglobin, Hb, g/L	133.5 ± 17.16	136.41 ± 13.4	0.42 ^1^
Platelets, PLT, 10^3^ µL	255.29 ± 58.66	249.52 ± 58.17	0.68 ^1^
*Hormones*			
Cortisol, nmol/L, mean ± SD	410 ± 143.2	417.2 ± 137.1	0.8 ^1^
Prolactin, ng/mL, Me (IQR)	17.2 (9.0–21.3)	15.9 (12.2–23.2)	0.4 ^2^

^1^ Student’s unpaired *t*-test; ^2^ Mann–Whitney U-test; ^3^ χ^2^ test. Significant differences are highlighted in bold. The hemogram and hormonal profile were analyzed using morning fasting blood samples taken the next morning after admission.

## Data Availability

All data generated or analyzed during this study are included in this published article. Primary datasets generated during and/or analyzed during the current study are available from the corresponding author on reasonable request.
